# Does the Conservative Non-pharmacological Management of Knee Osteoarthritis in Switzerland Reflect the Clinical Guidelines? A Survey Among General Practitioners, Rheumatologists, and Orthopaedic Surgeons

**DOI:** 10.3389/fresc.2021.658831

**Published:** 2021-06-14

**Authors:** Lea Ettlin, Irina Nast, Erika O. Huber, Karin Niedermann

**Affiliations:** ^1^School of Health Professions, Institute of Physiotherapy, Zurich University of Applied Sciences, Winterthur, Switzerland; ^2^Department of Health Sciences and Health Policy, University of Lucerne, Lucerne, Switzerland

**Keywords:** osteoarthritis management, conservative treatment, exercise, knee pain, primary care (MeSH)

## Abstract

**Introduction:** The International Guidelines recommend exercise, education and weight management (if appropriate) as the first-line conservative treatment for patients with knee osteoarthritis (OA) to enhance their self-management. The aim of this study was to investigate the current state of conservative non-pharmacological management of patients with knee OA in Switzerland and to explore the perceived barriers and facilitators to the application of the guideline recommendations.

**Materials and methods:** Eleven semi-structured interviews with selected general practitioners (GPs), rheumatologists and orthopaedic surgeons were performed. Based on these results, an online survey was developed and sent to the members of three scientific medical societies. Questions addressed the frequency of diagnostic measures, treatment options, reasons for referral to exercise and also barriers and facilitators.

**Results:** A total of 234 members responded. They indicated that patients normally present due to pain (*n* = 222, 98.2%) and functional limitations of the knee (*n* = 151, 66.8%). In addition to clinical assessment, X-ray (*n* = 214, 95.5%) and MRI (*n* = 70, 31.3%) were the most frequently used diagnostic measures. Treatment options usually involved patient education for diagnosis (*n* = 223, 98.6%) and suitable activities (*n* = 217, 96%), pharmacological treatment (*n* = 203, 89.8%) and referral to physiotherapy (*n* = 188, 83.2%). The participants estimated that they had referred 54% of their patients with knee OA for a specific exercise. The referral to exercise was driven by “patient expectation/high level of suffering” (*n* = 73, 37.1%) and their “own clinical experience” (*n* = 49, 24.9%). The specialists rated the most important barriers to referral to exercise as “disinterest of patient” (*n* = 88, 46.3%) and “physically active patient” (*n* = 59, 31.1%). As the most important facilitators, they rated “importance to mention exercise despite the short time of consultation” (*n* = 170, 89.4%) and “insufficiently physically active patient” (*n* = 165, 86.9%).

**Discussion:** A substantial evidence–performance gap in the management of patients with knee OA appears to exist in Switzerland. For the systematic referral to exercise as the first-line intervention, it might be useful for medical doctors to suggest a structured exercise programme to patients with knee OA, rather than just advising general exercise.

## Introduction

Osteoarthritis (OA) is one of the most prevalent musculoskeletal diseases in Switzerland, affecting around two million people (1). Of the world's population, one-fifth of people aged over 50 years and a half aged over 65 years are diagnosed with OA, particularly knee OA (2–4). The European League Against Rheumatism (EULAR) recommendations assert that the use of imaging in the clinical management of peripheral joint OA is not required for the diagnosis of patients presenting with typical symptomatic knee OA, such as suffering from pain and limitation of function (4). In clinical practise, however, diagnosis by clinical symptoms is often complemented by radiography and MRI. Meta-analyses have shown high-quality evidence for the positive effects of exercise on pain reduction and the quality of life of patients with knee OA (5–7). The clinical guidelines for the management of knee OA from the American College of Rheumatology (ACR), the EULAR and OA Research Society International (OARSI) recommend individual management that includes patient education and exercise, and also appropriate weight management (6, 8–10), to promote patient self-management as the first-line intervention. Education, exercise and appropriate weight control should be offered to all patients with knee OA. Some patients might require additional second-line treatment, for example, pharmacological therapy or passive treatment provided by therapists (6, 7, 9–11), but few patients (estimated at 10–15%) need an immediate surgery (12).

Knee OA is one of the most frequent diagnoses in Swiss hospitals (13, 14), but information on the conservative management prior to the surgery is sparse. Therefore, it is unclear whether, and to what extent, the guideline recommendations concerning exercise and education are being applied in conservative management (14). Joint pain and knee OA are generally managed in the primary care in many countries (2, 11, 15) and in Switzerland conservative treatment may also be taking place in primary care, since the first contact for patients with knee OA symptoms is usually their general practitioner (GP). All permanent Swiss residents pay compulsory health insurance covering a package of benefits for health care services, while including yearly deductibles and co-payments. The basic benefit package of compulsory health insurance is set by law and is the same for each insurance company throughout Switzerland. The insurance policy covers primary care from the licenced medical doctors and, after referral by a medical doctor, physiotherapeutic treatment. Swiss residents have a freedom of choice of providers and direct access (without a referral from a GP) to secondary care, that is medical specialists, unless they are enrolled in a managed care plan (16). Thus, patients also have direct access to rheumatologists and orthopaedic surgeons, who may also be involved in the management of patients with knee pain and knee OA.

Exercise and physical activity not only effectively prevent knee OA and diminish its progression (17), but are also important to society in general, given the challenges that the health care system is facing with increasing costs, an ageing population and an increase in obesity (11). Observations of the clinical practise suggest that despite high-quality evidence on the effectiveness and cost-effectiveness of exercise, conservative non-pharmacological treatment may be underused in Switzerland. Research has identified various barriers to the use of exercise as the first-line treatment, such as the trivialisation of OA as being inevitable, and the lack of incentives (18, 19). Furthermore, health care providers often have the perception that the effectiveness of exercise is limited, or the general public and patients lack information on its potential (20, 21). Moreover, a long-term adherence to exercise is challenging because it requires a behavioural change in patients and appropriate support from health care providers (22).

The main aim of this study was to investigate the current conservative management of patients with knee OA in Switzerland from the perspective of GPs, rheumatologists and orthopaedic surgeons and to evaluate what drives their decision-making on exercise and patient education as the first-line intervention, either for or against. Further aims were to analyse whether the preference for conservative treatment varies between three types of specialists (GPs, rheumatologists and orthopaedic surgeons) or whether there were dissimilarities in their decision-making. Additionally, it was an objective to explore possible differences between the views of the specialists regarding the barriers and facilitators to the application of the guideline recommendations.

## Materials and Methods

### Study Design

An exploratory sequential mixed method design was used to explore and evaluate the treatment options for the conservative management of patients with knee OA (23). Firstly, semi-structured telephone interviews were performed (qualitative element), followed by a cross-sectional online survey (quantitative element).

#### Semi-Structured Telephone Interviews

Eleven semi-structured interviews with GPs (*n* = 4), rheumatologists (*n* = 4) and orthopaedic surgeons (*n* = 3) were performed to determine the reasons for their patient consultations and which other diagnostic tools they generally used in addition to the clinical assessment and conservative treatment. The interviews were also performed to identify the reasons for referral to exercise and the related barriers and facilitators. The informants were selected based on the region, language area and gender. Interviews were conducted by telephone and lasted between 5 and 15 min. They were audiotaped and transcribed. An analysis was performed using a directed content analysis (24), that is according to the recommendations of clinical guidelines and literature in the field of the conservative management of knee OA.

#### Online Survey

The results from these interviews formed the basis for the survey questions. The final version of the survey contained 14 questions that encompassed the above-mentioned interview topics:

1. Consultation reasons and diagnostic tools: questions on the main symptoms presented by patients, assessed on a Likert scale from 3 (always) to 0 (never), and the use of diagnostic tools (in addition to the clinical assessment) established through multiple-answer options.
2. Treatment options in the conservative knee OA treatment: assessed on a Likert scale from 3 (always) to 0 (never).
3. Reasons for referral to exercise: participants were asked to prioritise five reasons from 1 to 5.
4. Barriers and facilitators for referral to exercise, as recommended by the guidelines: assessed on a Likert scale from 5 (I fully agree) to 1 (I disagree).

Additionally, enquiries were made on the rate (%) of referral of their patients with knee OA to exercise, along with an open question on indication criteria for referral to surgery. Six initial questions on the characteristics of the survey participants and their work with patients with knee OA patients were asked. After pilot testing with three medical specialists and three researchers, the survey was slightly modified to improve its clarity. The survey was kept as short as possible, that is <10 min for completion, to take into account the limited time of physicians busy with clinical work (24).

The GPs, rheumatologists and orthopaedic surgeons in the German, French and Italian language areas of Switzerland were invited to participate in the online survey. Three Swiss scientific medical societies supported the study by sending the survey link to their members: the association of Swiss family physicians and paediatricians (mfe, 4,242 members); the Swiss Society of Rheumatology (SGR, 570 members) and the Swiss Society of orthopaedics and traumatology (Swiss orthopaedics, 759 members). Retired persons and those who did not treat patients with knee OA (e.g., paediatricians) were also among the recipients. The members received links by an email to access a German, French or English version of the survey using the SurveyMonkey® (San Mateo, CA, USA). The SGR sent a reminder to their members after 3 weeks. The online survey was closed after 3 months.

### Statistical Analysis

Demographics and work characteristics of the survey participants are presented as frequencies and percentages or as means and SD, as appropriate. A subgroup analysis of the survey responders was performed between GPs, rheumatologists and orthopaedic surgeons. For representativeness, the Levene's tests (one-factor ANOVA) were performed to ascertain whether there were differences in gender between the responders and non-responders (members of the associations) among each subgroup. The geographical distribution was analysed by the frequency of responders from each language region and each subgroup.

Appropriately, a variance analysis (one-factor ANOVA) was applied to test the significance of differences between the subgroups. In addition, the Likert scales were dichotomised for group comparison, that is the answer options “always/often” and the answer options “seldom/never” and, additionally, the answer option “I don't know.”

Statistics were performed using the IBM SPSS software, version 25 (IBM SPSS, Armonk, NY, USA).

For the open question on indication criteria for surgery, the answers were coded into the most repeated topics and their frequencies analysed.

### Ethical Approval

This survey did not fall within the scope of the Swiss Human Research Act, and thus authorisation from an ethics committee was not required. The survey participants received the invitation directly from their societies. The members of the research group had no direct access to the participants. Furthermore, the survey link was not personalised, and the survey did not track the' IP-addresses of the participants in order to preserve anonymity. Respondents were informed on the starting page that their participation automatically provided their informed consent.

## Results

### Characteristics of Participants

A total of 5,571 specialists from the three medical societies were invited to participate in the survey and, of these, 234 (4.4%) responded, with a response rate of 1.8% from GPs, 14.7% from rheumatologists and 9.2% from orthopaedic surgeons. On a language basis, 78% of the participants responded in German, 18% in French and 4% in English. Responders were included for the analysis when they answered at least the three questions concerning consultation reasons, diagnostic measures and treatment options in the conservative knee OA management in addition to the demographic questions. This resulted in 226 specialist responses for the analysis, of which 72 were from GPs, 84 from rheumatologists and 70 from orthopaedic surgeons. Their characteristics are reported in Table 1.

**Table 1:** Characteristics of the survey participants (*n* = 226).

	**All** ** (*n* = 226)**	**GPs** ** (*n* = 72)**	**Rheumatologists (*n* = 84)**	**Orthopaedic surgeons** ** (*n* = 70)**
Female, *n* (%)	53 (23.5)	30 (41.7)	20 (23.8)	3 (4.3)
Clinical experience, years	21.02 (10.2)	23.6 (11.3)	21.9 (9.5)	17.3 (8.9)
Level of employment (%)	83.9 (30.2)	71.5 (31.3)	82.6 (29.0)	98.2 (24.0)
Self-reported number of knee OA diagnoses performed per month	13.5 (22.2)	3.8 (3.8)	11.1 (13.6)	26.6 (32.8)
Self-reported number of patients with knee OA treated per month	20.1 (25.7)	11.0 (17.5)	19.1 (20.0)	30.5 (33.7)

*Values are means (SD) unless stated otherwise*.

*Level of employment: reported % hours of 100% working hours/week*.

### Survey Results

#### Consultation Reasons and Diagnostic Tools Used in Addition to Clinical Assessment

Pain in the knee joint (*n* = 222, 98.2%) and limited function (*n* = 151, 66.8%) were reported as being the main reasons for patients to consult a specialist. The rate of patients with ‘referrals from other doctors' varied substantially between the specialist subgroups, from 2.8% for GPs to 91.5% for orthopaedic surgeons (Table 2).

**Table 2:** Main reasons for patients to seek medical advice: from the perception of the specialists (*n* = 226).

		**Always**	**Often**	**Seldom**	**Never**
Pain in the knee joint	All	141 (62.4)	81 (35.8)	3 (1.3)	1 (0.4)
	GPs	50 (69.4)	22 (30.6)	0	0
	Rheum.	51 (60.7)	29 (34.5)	3 (3.6)	1 (1.2)
	Orthop.	40 (57.1)	30 (42.9)	0	0
Limited function of the knee joint	All	9 (4.0)	142 (62.8)	71 (31.4)	4 (1.8)
	GPs	2 (2.8)	43 (59.7)	27 (37.5)	0
	Rheum.	3 (3.6)	57 (67.9)	21 (25.0)	3 (3.6)
	Orthop.	4 (5.7)	42 (60.0)	23 (32.9)	1 (1.4)
Referral from other medical doctors	All	18 (8.0)	114 (50.4)	34 (15.0)	60 (26.5)
	GPs	0	2 (2.8)	12 (16.7)	58 (80.6)
	Rheum.	9 (10.7)	57 (67.9)	17 (20.2)	1 (1.2)
	Orthop.	9 (12.9)	55 (78.6)	5 (7.1)	1 (1.4)
Stiffness of the knee joint	All	7 (3.1)	98 (43.4)	118 (52.2)	3 (1.3)
	GPs	3 (4.2)	31 (43.1)	37 (51.4)	1 (1.4)
	Rheum.	1 (1.2)	43 (51.2)	38 (45.2)	2 (2.4)
Orthop.	3 (4.3)	24 (34.3)	43 (61.4)	0	
Because of another diagnosis	All	6 (2.7)	79 (35.0)	131 (58.0)	10 (4.4)
	GPs	1 (1.4)	29 (40.3)	41 (56.9)	1 (1.4)
	Rheum.	4 (4.8)	41 (48.8)	37 (44.0)	2 (2.4)
	Orthop.	1 (1.4)	9 (12.9)	53 (75.7)	7 (10.0)

*Values are absolute and relative frequencies*.

*All. All participants (n = 226)*.

*GPs. General practitioners (n = 72)*.

*Rheum. Rheumatologists (n = 84)*.

*Orthop. Orthopaedic surgeons (n = 70)*.

**Figure 1:**
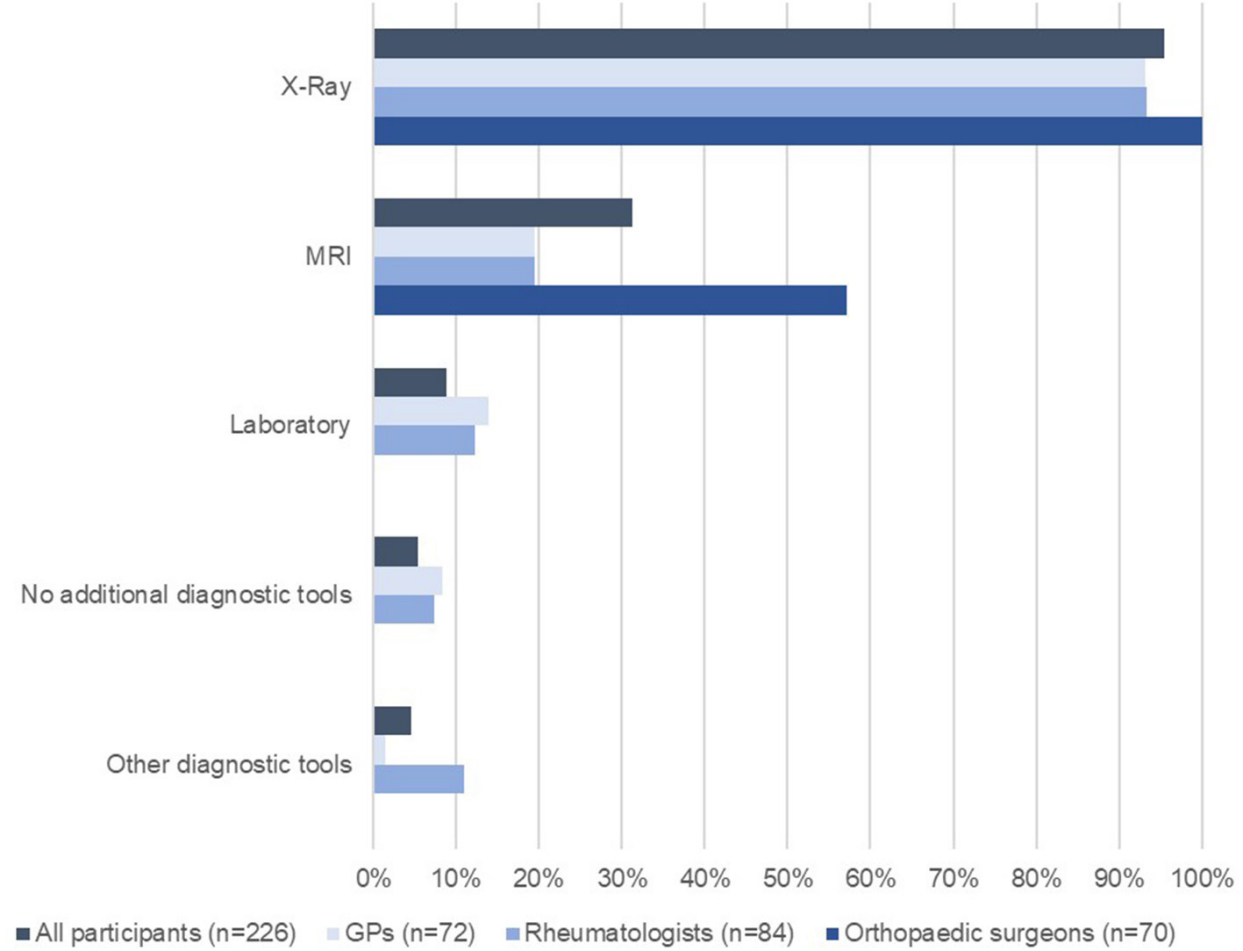
Diagnostic tools used to complement clinical assessment when clinical signs indicated knee OA (*n* = 226).

Figure 1 displays the use of multiple additional diagnostic tools when clinical signs indicated knee OA. Irrespective of the medical discipline, an X-ray (*n* = 214; 95.5%) was the most frequently used diagnostic tool to confirm the clinical diagnosis. An MRI was used substantially less (*n* = 70; 31.3%) and mainly by orthopaedic surgeons (*n* = 40; 57.1%).

#### Conservative Treatment Options

The conservative treatment options most mentioned by all participants were “informing the patients about the diagnosis” (*n* = 223; 98.6%), “recommending suitable activities or sports” (*n* = 217; 96%), “pharmacological treatment” (*n* = 203; 89.8%) and “referral to physiotherapy” (*n* = 188; 83.2%). There were no differences between the subgroups for any of these treatment options (*p* = 0.056). The only difference between the subgroups was for “referral to another medical health specialist.” Of the GPs, 26.6% “always” or “often” made referrals, compared to 6% of the rheumatologists and 10% of the orthopaedic surgeons (*p* < 0.001) (Table 3).

**Table 3:** Conservative treatment options the medical specialists use for their patients after diagnosing knee OA (*n* = 226).

		**Always**	**Often**	**Seldom**	**Never**
Patient education/Informed patient (diagnosis)	All	215 (95.1)	8 (3.5)	1 (0.4)	2 (0.9)
	GPs	68 (94.4)	2 (2.8)	1 (1.4)	1 (1.4)
	Rheum.	81 (96.4)	2 (2.4)	0	1 (1.2)
	Orthop.	66 (94.3)	4 (5.7)	0	0
Instruction: suitable activities/sports	All	153 (67.7)	64 (28.3)	8 (3.5)	1 (0.4)
	GPs	46 (63.9)	20 (27.8)	6 (8.3)	0
	Rheum.	61 (72.6)	21 (25.0)	1 (1.2)	1 (1.2)
	Orthop.	46 (65.7)	23 (32.9)	1 (1.4)	0
Pharmacological treatment	All	52 (23.0)	151 (66.8)	21 (9.3)	2 (0.9)
	GPs	13 (18.1)	51 (70.8)	8 (11.1)	0
	Rheum.	23 (27.4)	55 (65.5)	5 (6.0)	1 (1.2)
	Orthop.	16 (22.9)	45 (64.3)	8 (11.4)	1 (1.4)
Referral to physiotherapist	All	40 (17.7)	148 (65.5)	35 (15.5)	3 (1.3)
	GPs	11 (15.3)	51 (70.8)	9 (12.5)	1 (1.4)
	Rheum.	19 (22.6)	55 (65.5)	9 (10.7)	1 (1.2)
	Orthop.	10 (14.3)	42 (60.0)	17 (24.3)	1 (1.4)
Instruction to perform specific exercises (e.g., strength exercises)	All	79 (35.0)	107 (47.3)	37 (16.4)	3 (1.3)
	GPs	23 (31.9)	31 (43.1)	17 (23.6)	1 (1.4)
	Rheum.	33 (39.3)	38 (45.2)	11 (13.1)	2 (2.4)
	Orthop.	23 (32.9)	38 (54.3)	9 (12.9)	0
Instruction: weight reduction	All	74 (32.7)	108 (47.8)	41 (18.1)	3 (1.3)
	GPs	27 (37.5)	33 (45.8)	11 (15.3)	1 (1.4)
	Rheum.	30 (35.7)	37 (44.0)	15 (17.9)	2 (2.4)
	Orthop.	17 (24.3)	38 (54.3)	15 (21.4)	0
Load removal through crutches/canes, orthosis	All	3 (1.3)	40 (17.7)	159 (70.4)	24 (10.6)
	GPs	2 (2.8)	13 (18.1)	49 (68.1)	8 (11.1)
	Rheum.	1 (1.2)	15 (17.9)	61 (72.6)	7 (8.3)
	Orthop.	0	12 (17.1)	49 (70.0)	9 (12.9)
Referral to other medical health specialist	All	2 (0.9)	29 (12.8)	159 (70.4)	36 (15.9)
	GPs	2 (2.8)	17 (23.6)	53 (73.6)	12 (14.3)
	Rheum.	0	5 (6.0)	67 (79.8)	24 (34.3)
	Orthop.	0	7 (10.0)	39 (55.7)	0

The 226 participants estimated that they referred 54% (SD 27.8) of their patients with knee OA to exercise treatment. The subgroup analysis revealed no significant differences in the estimated referral to specific exercise (*p* = 0.058).

#### Reasons for Referral to Specific Exercise

**Figure 2:**
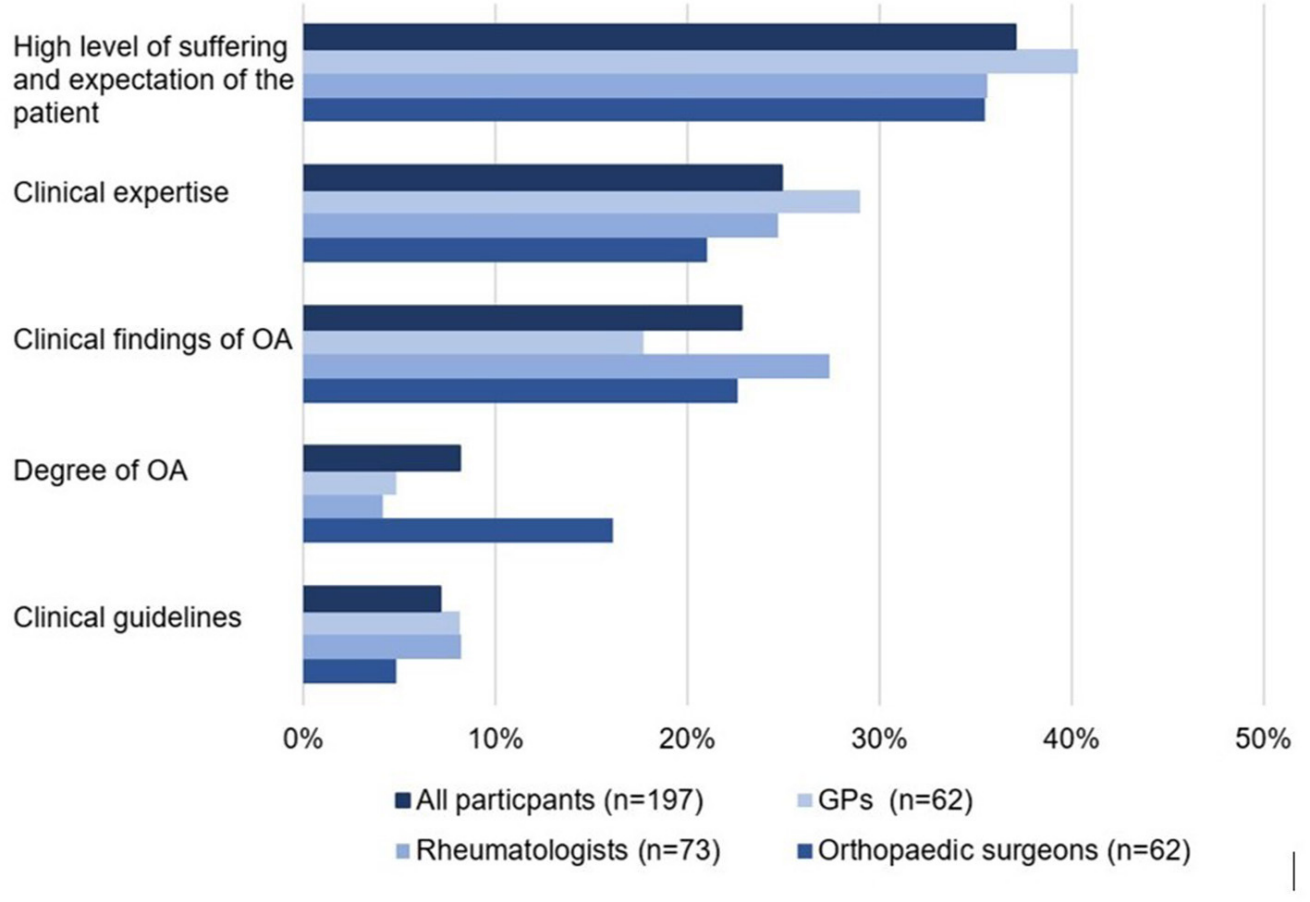
Ranking of the highest prioritisation of five rated reasons for the referral to exercise (*n* = 197).

The participants prioritised five reasons that influenced their decision-making for referring patients to exercise. Figure 2 displays the frequencies of the highest-rated prioritisation of the reasons in a ranked order. “High level of suffering and expectation of the patient” was placed in the first rank as the highest-rated prioritisation that influences the referral to exercise, followed by “clinical expertise,” “clinical picture of OA” and “degree of OA,” whereas “clinical guidelines” was placed last. The subgroup analysis showed significant (*p* = 0.008) differences only for the reason “degree of OA.” Orthopaedic surgeons rated the “degree of OA” as the most important reason for referral to exercise compared to rheumatologists or GPs.

#### Barriers and Facilitators for Referral to Exercise

The barriers and facilitators for referring patients with knee OA to exercise, as recommended by the guidelines, are displayed in Table 4.

**Table 4:** Barriers and facilitators for applying clinical guideline recommendations, that is reasons to (not) enforce exercise (*n* = 190).

		**Item seen as a barrier**					**Item seen as a facilitator**				
**Item**		**Fully agree**	**Partly agree**	**I don't know**	**Partly disagree**	**Disagree**	**Fully agree**	**Partly agree**	**I don't know**	**Partly disagree**	**Disagree**
Priority to mention exercise in the short time of consultation	All	3 (1.6)	14 (7.4)	10 (5.3)	78 (41.1)	85 (44.7)	100 (52.6)	70 (36.8)	5 (2.6)	10 (5.3)	5 (2.6)
	GPs	1 (1.6)	5 (8.2)	5 (8.2)	25 (34.7)	25 (34.7)	30 (49.2)	26 (42.6)	3 (4.9)	1 (1.6)	1 (1.6)
	Rheum.	0	5 (7.0)	3 (4.2)	25 (35.2)	38 (53.5)	45 (63.4)	21 (29.6)	2 (2.8)	0	3 (4.2)
	Orthop.	2 (3.4)	4 (6.9)	2 (3.4)	28 (48.3)	22 (37.9)	25 (43.1)	23 (39.7)	0	9 (15.5)	1 (1.7)
Availability of information on exercise	All	1 (0.5)	26 (13.7)	15 (7.9)	86 (45.3)	62 (32.6)	37 (19.5)	79 (41.6)	25 (13.2)	45 (23.7)	4 (2.1)
	GPs	1 (1.6)	9 (14.8)	6 (9.8)	29 (47.5)	16 (26.2)	11 (18.0)	23 (37.7)	11 (18.0)	15 (24.6)	1 (1.6)
	Rheum.	0	7 (9.9)	3 (4.2)	29 (40.8)	32 (45.1)	21 (29.6)	24 (33.8)	8 (11.3)	15 (21.1)	3 (4.2)
	Orthop.	0	10 (17.2)	6 (10.3)	28 (48.3)	14 (24.1)	5 (8.6)	32 (55.2)	6 (10.3)	15 (25.9)	0
Doubt about exercise's benefit/Benefit of exercise as evidence based	All	1 (0.5)	18 (9.5)	8 (4.2)	68 (35.8)	95 (50.0)	73 (38.4)	77 (40.5)	22 (11.6)	13 (6.8)	5 (2.6)
	GPs	0	5 (8.2)	2 (3.3)	23 (37.7)	31 (50.8)	23 (37.7)	23 (37.7)	12 (19.7)	2 (3.3)	1 (1.6)
	Rheum.	1 (1.4)	3 (4.2)	4 (5.6)	19 (26.8)	44 (62.0)	35 (49.3)	28 (39.4)	3 (4.2)	2 (2.8)	3 (4.2)
	Orthop.	0	10 (17.2)	2 (3.4)	26 (44.8)	20 (34.5)	15 (25.9)	26 (44.8)	7 (12.1)	9 (15.5)	1 (1.7)
(Dis-)Interest of patient	All	13 (6.8)	75 (39.5)	25 (13.2)	56 (29.5)	21 (11.1)	33 (17.4)	113 (59.5)	19 (10.0)	21 (11.1)	4 (2.1)
	GPs	4 (6.6)	19 (31.1)	8 (13.1)	24 (39.3)	6 (9.8)	13 (18.1)	33 (54.1)	8 (13.1)	6 (9.8)	1 (1.6)
	Rheum.	8 (11.3)	31 (43.7)	8 (11.3)	16 (22.5)	8 (11.3)	14 (19.7)	40 (56.3)	6 (8.5)	8 (11.3)	3 (4.2)
	Orthop.	1 (1.7)	25 (43.1)	9 (15.5)	16 (27.6)	7 (12.1)	6 (10.3)	40 (69.0)	5 (8.6)	7 (12.1)	0
Patient is (not) sufficiently physically active	All	7 (3.7)	52 (27.4)	14 (7.4)	90 (47.4)	27 (14.2)	41 (21.6)	124 (65.3)	13 (6.8)	10 (5.3)	2 (1.1)
	GPs	1 (1.6)	14 (23.0)	5 (8.2)	34 (55.7)	7 (11.5)	13 (21.3)	41 (67.2)	2 (3.3)	4 (6.6)	1 (1.6)
	Rheum.	4 (5.6)	16 (22.5)	7 (9.9)	31 (43.7)	13 (18.3)	20 (28.2)	45 (63.4)	4 (5.6)	1 (1.4)	1 (1.4)
	Orthop.	2 (3.4)	22 (37.9)	2 (3.4)	25 (43.1)	7 (12.1)	8 (13.8)	38 (65.5)	7 (12.1)	5 (8.6)	0
Applicability of guideline recommendations for exercise	All (*n* = 190)	3 (1.6)	15 (7.9)	50 (26.3)	71 (37.4)	51 (26.8)	38 (20.0)	83 (43.7)	42 (22.1)	20 (10.5)	7 (3.7)
	GPs	1 (1.6)	2 (3.3)	22 (36.1)	20 (32.8)	16 (26.2)	12 (19.7)	28 (45.9)	16 (26.2)	3 (4.9)	2 (3.3)
	Rheum.	1 (1.4)	5 (7.0)	15 (21.1)	26 (36.6)	24 (33.8)	18 (25.4)	36 (50.7)	7 (9.9)	7 (9.9)	3 (4.2)
Orthop.	1 (1.7)	8 (13.8)	13 (22.4)	25 (43.1)	11 (19.0)	8 (13.8)	19 (32.8)	19 (32.8)	10 (17.2)	2 (3.4)	

*Values are absolute and relative frequencies*.

*All. All participants (n = 190)*.

*GPs. General practitioners (n = 61)*.

*Rheum. Rheumatologists (n = 71)*.

*Orthop. Orthopaedic surgeons (n = 58)*.

The most important perceived barriers were “disinterest of patient” (46.3%) and “already physically active patient” (31.1%). Of the GPs, 19.7% chose the answer option “I don't know” if the “effectiveness of exercise is evidence based” represents a barrier, compared to 4.2% of the rheumatologists and 12.1% of the orthopaedic surgeons. The answer option “I don't know” was also chosen for the “feasibility of the guideline recommendations to suggest exercise” by 36.1% of the GPs, 21.1% of the rheumatologists and 22.4% of the orthopaedic surgeons. The most often rated facilitators were “priority to mentioning exercise in the short time of a consultation” (*n* = 170; 89.4%) and “insufficiently physically active patient” (*n* = 165; 86.9%). Furthermore, the “guideline recommendations” (*n* = 121; 63.7%) and “anticipated/perceived interest of patients” (*n* = 146; 76.9%) were stated as facilitating factors to suggest exercise to patients.

#### Indication Criteria for Referral to Surgery (n = 226)

The most frequently mentioned criteria for referral to surgery were “high level of pain and suffering” (*n* = 142; 62.8%), “exhaustion of conservative treatment strategies” (*n* = 106; 46.9%) and “limitation of functioning in ADL” (*n* = 70; 31%). There was often a combination of two or more criteria for referral to surgery. The subgroup analysis showed differences in the main criteria for the referral to surgery, that is “high level of pain and suffering” was the main criteria for the GPs (65.3%) and rheumatologists (67.9%), whereas “exhaustion of conservative treatment strategies” was the main criteria for the orthopaedic surgeons (57.1%).

## Discussion

To our knowledge, this is the first study to survey the conservative management of patients with knee OA in Switzerland. This study showed that the main symptoms of knee OA for most patients are pain and limited function. X-ray and MRI were often used to confirm the diagnosis when clinical signs indicated knee OA. Differences between groups of the three medical specialists were found between the use of diagnostics tools and referral to other medical specialists. The treatment options that were applied were similar for groups of the three medical specialists. They used patient education for the diagnosis and the treatment options, such as instructions on exercise, pharmacological treatment and referral to physiotherapy treatment. Another important finding was that the estimated referral rate to exercise was only about 54%. The finding that the guideline recommendations were not systematically applied in Switzerland is conclusive. Some important barriers and facilitators to adherence to the guidelines were detected in the semi-structured interviews and evaluated in the online survey. “Disinterest of the patient” and “already physically active patient” were rated as the most important barriers, whereas the “importance of mentioning exercise despite the short time of consultation” and patients who were “insufficiently physically active” were rated as the most important facilitators.

The surveyed GPs, rheumatologists and orthopaedic surgeons reported that they frequently used X-ray and MRI in addition to their clinical assessments. The guideline recommendations suggest that a careful clinical examination is sufficient unless there are any additional benefits to patients of using imaging tools as part of the diagnostic pathway, or to confirm a difference in diagnosis (11, 25, 26). The fact that orthopaedic surgeons showed a substantially higher use of MRI could be due to referrals from other specialists, and that they use MRI results as part of their decision-making process to decide whether surgery is indicated or not. Those knee OA cases that are referred for evaluation of the surgical option are also probably the most severe.

There is a gap between the ratings of participants for the treatment options, especially regarding “referral to physiotherapy” and their estimated rate of “referrals to specific exercise.” More than 80% of the specialists chose “referral to physiotherapy” as a treatment option, whereas the estimated rate of “referrals to specific exercise” for all subgroups was only about 54%. The estimated referral rate to specific exercise could be interpreted as including any exercise or as a referral to physiotherapy. Decision-making for referral to exercise in all subgroups was driven by “patient expectation,” “high level of suffering” and their “own clinical experience” and the clinical findings of the OA. Interestingly, although orthopaedic surgeons are the last specialists consulted when surgery is indicated, they did not show a lower rate of referral to exercise. They also prioritised “degree of OA” higher than GPs or rheumatologists as a reason for referral to exercise. It can be assumed that orthopaedic surgeons refer patients with lower degrees of OA to exercise and patients with higher degrees of OA to surgery.

In Switzerland, the guidelines are not issued by a national institution. Medical societies often publish discipline-specific guidelines on their websites. However, there is a great difference in the quality of the published guidelines (27). In our survey, the three specialist medical groups were generally conversant with the guideline recommendations for knee OA. Nevertheless, there are some barriers to the use of the guidelines, for example, the guidelines may be too rigid to apply to individual patients, or they may be a challenge to the autonomy of the medical doctor (28). Although GPs are most often challenged with multimorbid patients, a situation where the guideline recommendations are often not systematically applicable (29), they did not show a lower adherence to the guidelines in this survey. It should be noted that Switzerland, regarding financing, organisation and provision of health care, has a decentralised system. The economic status of the patient does not usually preclude access to care and, as a result, there is no restriction on surgery. There was a significant difference in this survey in the referral pattern for referring or treating referred patients by GPs, rheumatologists and orthopaedic surgeons. GPs are usually the first point of contact on the treatment pathway and may refer patients to rheumatologists and orthopaedic surgeons who, as a consequence, treat more referred patients than direct access patients. Nevertheless, it remains unclear as to whether patients were referred to orthopaedic surgeons for potential surgery before or after the exhaustion of conservative treatment.

The evidence on the effectiveness of exercise in reducing pain, improving physical function and raising the quality of life of people with knee OA in the short and long terms, has been confirmed repeatedly in meta-analyses (5, 9, 15, 30). As early as 2015, the Cochrane Collaboration stated that the evidence for the effects of the exercise was so convincing that further studies were unlikely to change this strong and high quality of evidence (5). Previous studies had shown that suboptimal use of exercise could be due to preferences of patients or lack of information on the conservative treatment options (19, 31). The surveyed specialists have an important impact, depending on their own attitudes and how they communicate the possible treatment options. They need to be aware that the motivation of a patient to exercise is enhanced by explaining the positive outcomes of exercise and by giving their support to the shared decision to exercise (9, 19, 31). To facilitate the application of guidelines and referral to exercise into clinical practise, it might be useful to translate the recommendations into a best-practise, high-quality, exercise programme and provide easy guidance instructions for both patients and health care providers. Structured exercise and education programmes for knee OA have been successfully developed and established throughout the world, for example “Osteoarthritis Chronic Care Program (OACCP), Australia,” “Better management of patients with osteoarthritis (BOA), Sweden,” “Good Life with osteoarthritis in Denmark (GLA:D),” “Osteoarthritis Healthy Weight For Life (OA HWFL), Australia,” “Amsterdam osteoarthritis cohort (AMSOA), the Netherlands” or “Joint implementation of osteoarthritis guidelines in the West Midlands, UK (JIGSAW)” (11). All these programmes, endorsed by OARSI, translate the guideline recommendations into practise with the goal of enhancing patient self-management. The existing programmes deliver the first-line exercise and education treatment with varying degrees of intensity and standardisation. Some programmes include support for weight management. The content of many programmes is similar, but they target different groups of patients and health care professionals. Most programmes focus on pain, function and quality of life. Establishing a programme may be challenging due to financial restrictions and, possibly even more importantly, taking into consideration the individual interests of all the stakeholders involved. However, such a structured exercise and education programme could be an effective way of overcoming the evidence–performance gap in Switzerland and supporting referral to exercise. Moreover, it would support the national strategy 2017–2024 for non-communicable diseases (NCDs) (including musculoskeletal diseases) that emphasises systematic disease management and patient self-management (32). Knee OA is no longer seen as a “bone to bone” disease caused by “wear and tear” that necessitates quick (surgical) action. It is rather understood to be a long-term illness affecting the entire person that requires effective management of the symptoms. Understanding the disease, its causes and consequences may enhance the motivation of the patient to exercise and subsequent self-management (18). For the successful implementation of a best-practise exercise and education programme as the first-line treatment, the barriers and facilitators identified by the surveyed specialists need to be considered, for example, the interest of patients in exercise or physical activity (33).

This study has some limitations. The response rate was relatively low and potentially biassed, since the survey was sent to all members of the three scientific medical societies, some of whom did not work with patients with knee OA, and therefore did not participate in the survey, for example, orthopaedic surgeons for children or general paediatricians. Furthermore, medical doctors are invited very often to participate in surveys and, due to lack of time or interest, cannot respond to all invitations (34). Regarding our survey, since the participants have patients with many health problems, the topic of conservative knee OA management was presumably not their main focus. The survey focused on the conservative management of knee OA, specifically the use of exercise as a first-line treatment, because of the perception of it being an underused treatment option in Switzerland, despite being recommended as a core treatment by the International Guidelines. The specialists invited to participate in the survey, however, may really have been more interested in exercise as one of several therapeutic options and hence not participated in the survey. This could have resulted in self-selection bias, with the consequence that only persons interested in the topic participated (35). However, with regard to profession, gender and area distribution, the participants were representative of the members of their societies. Since all members of the three societies were invited, regardless of whether they worked with patients with knee OA, the response rate pertaining to non-respondent bias and self-selection bias can be considered acceptable (21, 36, 37). A further limitation may have led to choice bias or position bias, due to the multiple-choice questions and the technically imposed need to choose one of the answer options to progress further with the survey (18).

## Conclusion

The conservative non-pharmacological management of patients with knee OA is comparable between GPs, rheumatologists and orthopaedic surgeons concerning diagnostics and the use of treatment options. However, the respondents to our survey reported that only 54% of their patients were referred to a structured exercise. The guideline recommendations do not seem to have been systematically applied by any of the specialists. The systematic referral to exercise as the first-line intervention is not occurring. We conclude that there is substantial evidence–performance gap in the conservative non-pharmacological management of patients with knee OA in Switzerland. One proposed solution to overcome this gap is to translate the guideline recommendations into a structured exercise and education programme that can be systematically applied in clinical practise. A positive attitude of the referring medical doctors is key to the successful implementation of such a programme. It is important that medical doctors understand that not only is general exercise beneficial, but that specific exercise is the most effective treatment for patients with knee OA. For the successful implementation of exercise as the first-line intervention for knee OA, through a best-practise exercise and education programme, the barriers and facilitators need to be addressed.

## Data Availability Statement

The raw data supporting the conclusions of this article will be made available by the authors, without undue reservation.

## Ethics Statement

Ethical review and approval was not required for the study on human participants in accordance with the local legislation and institutional requirements. Written informed consent for participation was not required for this study in accordance with the national legislation and the institutional requirements.

## Author Contributions

LE and KN conceived and designed the study. LE collected and analyzed the data. LE, KN, IN, and EH contributed to the drafting and revision of the manuscript. All authors have read and approved the manuscript.

## Conflict of Interest

The authors declare that the research was conducted in the absence of any commercial or financial relationships that could be construed as a potential conflict of interest.
