# Supplementary material for: Does the Conservative Non-pharmacological Management of Knee Osteoarthritis in Switzerland Reflect the Clinical Guidelines? A Survey Among General Practitioners, Rheumatologists, and Orthopaedic Surgeons
Source: Front Rehabil Sci. 2021 Jun 14;2:658831. doi: 10.3389/fresc.2021.658831 (PMC9397950; doi:10.3389/fresc.2021.658831)
Supplement: Supplementary file 1 [file Data_Sheet_1.PDF]

Willkommen zur ZHAW Umfrage

Wir möchten den konservativen Behandlungspfad bei Kniearthrose erheben.

Wir danken Ihnen herzlich, dass Sie uns bei dieser Umfrage unterstützen!

Für das Ausfüllen des Fragebogens benötigen Sie ca. 7-8 Minuten.

Wir behandeln Ihre Antworten vertraulich und anonym!

Wenn Sie auf den "Weiter"-Knopf drücken, stimmen Sie zu, dass wir Ihre Antworten anonymisiert verwenden dürfen.

Demografisches/ Zu Ihrer Person

* 1. Was ist Ihr Beruf?

- ○ Rheumatologin/Rheumatologe
- ○ Orthopädin/Orthopäde
- ○ Hausärztin/Hausarzt
- ○ Sonstiges (bitte angeben)

* 2. In welchem Jahr haben Sie Ihren Facharzttitel erworben?

kein Facharzttitel, sondern (bitte nennen):

* 3. Wieviel Prozent arbeiten Sie?

4. Wie viele Diagnosen "Kniearthrose" stellen Sie durchschnittlich (pro Monat)?

5. Wie viele bereits diagnostizierte Kniearthrosepatienten behandeln durchschnittlich Sie wegen der Arthrose (pro Monat)?

Leitsymptomatik

6. Wegen welchen Gründen sind die Patientinnen/Patienten, die mit Kniearthrose diagnostiziert wurden, zu Ihnen gekommen?

	immer	häufig	selten	nie
Schmerz im Kniegelenk	○	○	○	○
Steifigkeit im Kniegelenk	○	○	○	○
Einschränkung ROM/Gelenksfunktion	○	○	○	○
Wegen einer anderen Diagnose	○	○	○	○
Überweisung von anderen Ärzten	○	○	○	○

Sonstiges (bitte angeben)

[ ]

Diagnostik

* 7. Welche der folgenden Massnahmen nutzen Sie für die Diagnosestellung, wenn die klinischen Zeichen für eine Kniearthrose sprechen?

- ○ Röntgen
- ○ MRI
- ○ Labor
- ○ keine der oben genannten
- ○ Andere (bitte nennen)

Konservative Behandlungsstrategien

8. Welche konservativen Behandlungsoptionen wenden Sie nach der Diagnosestellung an?

	Immer	häufig	Selten	Nie
Patienteninformation / Aufklärung Diagnose	○	○	○	○
Instruktion Selbstmanagement (geeignete Aktivitäten/Sportarten)	○	○	○	○
Instruktion spezifischer, therapeutischer Übungen	○	○	○	○
Instruktion Gewichtsreduktion	○	○	○	○
Medikamentöse Behandlung	○	○	○	○
Überweisung zur Physiotherapie	○	○	○	○
Überweisung zu anderer Fachärztin/anderem Facharzt	○	○	○	○
Entlastung durch Stöcke, Orthesen	○	○	○	○

Entscheidungshilfe

* 9. Es gibt klare Evidenz für das gezielte Kraft- und Gleichgewichtstraining zur Reduktion von Schmerz und der Verbesserung der Funktionsfähigkeit bei Kniearthrose.
Aus welchen Gründen überweisen Sie Kniearthrosepatieninnen/-patienten für dieses gezielte Training? Bitte priorisieren Sie (1 = höchste Priorität):

- [ ] Leidensdruck, Erwartungshaltung des Patienten
- [ ] Klinische Expertise / frühere Erfahrungswerte
- [ ] Klinische Guidelines
- [ ] Klinisches Bild der Arthrose
- [ ] Stadium der Arthrose

10. Wieviel Prozent (%) Ihrer Kniearthrose-Patienten überweisen Sie für ein solches Kraft- und Gleichgewichtstraining?

[ ]

klinische Guidelines / Leitlinien

Wir gehen davon aus, dass es förderliche als auch hinderliche Gründe gibt, Patienten in ein solches gezieltes Kraft- und Gleichgewichtstraining zu überweisen.
Die nächsten zwei Fragen beziehen sich deshalb auf solche Förderfaktoren und Barrieren.
Bitte beantworten Sie sie unabhängig voneinander

11. Mögliche förderliche Gründe: Sind die nachfolgend genannten Aspekte für Sie Gründe, um das gezielte Kraft- und Gleichgewichtstraining bei Ihren Patienten zu fördern?

	ja, voll und ganz	eher ja	weiss nicht	eher nein	nein, gar nicht
Spezifisches Training ist für mich ein genügend wichtiges Thema, um es in der knapp bemessenen Sprechstundenzeit anzusprechen	○	○	○	○	○
Ich habe ausreichende Information über das spezifische Training	○	○	○	○	○
Der Nutzen vom Kraft- und Gleichgewichtstraining bei Kniearthrosepatienten ist wissenschaftlich hinreichend belegt	○	○	○	○	○
Antizipiertes oder wahrgenommenes Interesse von Patient/in	○	○	○	○	○
Die Patientin / der Patient bewegt sich zu wenig	○	○	○	○	○
Guidelines empfehlen Kraft- und Gleichgewichtstraining	○	○	○	○	○

Gibt es für Sie andere Gründe, Kniearthrosepatienten zum Training zu motivieren?

[ ]

12. Mögliche hinderliche Gründe: Sind die nachfolgend genannten Aspekte für Sie Gründe, um das gezielte Kraft- und Gleichgewichtstraining bei Ihren Patienten NICHT zu fördern?

	ja, voll und ganz	eher ja	weiss nicht	eher nein	nein, gar nicht
Spezifisches Training hat für mich keine Priorität in der knapp bemessenen Sprechstundenzeit	○	○	○	○	○
Ich habe zu wenig Information über das spezifische Training	○	○	○	○	○
Ich habe Zweifel am Nutzen vom Kraft- und Gleichgewichtstraining bei Kniearthrosepatienten	○	○	○	○	○
Antizipiertes oder wahrgenommenes Desinteresse des Patienten	○	○	○	○	○
Die Patientin / der Patient bewegt sich ohnehin schon ausreichend	○	○	○	○	○
Die Guidelinesempfehlungen (Kraft- und Gleichgewichtstraining) sind zu abstrakt und nicht anwendbar	○	○	○	○	○

Gibt es für Sie andere Gründe, Kniearthrosepatienten NICHT zum Training zu motivieren?

Operative Behandlung

* 13. Wann ist für Sie eine operative Behandlung indiziert?

Herzlichen Dank

Wir danken Ihnen herzlich für Ihre Teilnahme an unserer Umfrage. Ihr Feedback ist uns sehr wichtig.
Falls Sie uns noch etwas mitteilen möchten, können Sie dies in unten stehendem Textfeld gerne tun.

Klicken Sie bitte auf >>Fertig<<, um die Eingabe Ihrer Antworten abzuschliessen.

14. Weiterer Kommentar:
